# OptimalMe Program: A Mixed Method Investigation into the Engagement and Acceptability of a Preconception Digital Health Lifestyle Intervention with Individual Coaching for Women’s Health and Behaviour Change

**DOI:** 10.3390/nu16050572

**Published:** 2024-02-20

**Authors:** Bonnie R. Brammall, Rhonda M. Garad, Helena J. Teede, Susanne E. Baker, Cheryce L. Harrison

**Affiliations:** Monash Centre for Health Research and Implementation, Monash University and Monash Health, Melbourne, VIC 3168, Australia; bonnie.brammall@monash.edu (B.R.B.); rhonda.garad@monash.edu (R.M.G.); helena.teede@monash.edu (H.J.T.); susanne.baker@monash.edu (S.E.B.)

**Keywords:** preconception, pregnancy, women’s health, lifestyle, nutrition, physical activity, digital health, health coaching, engagement, acceptability

## Abstract

Preconception interventions, specifically addressing general health, lifestyle behaviours and weight management, are limited despite their importance in optimising women’s health. The objective of this study is to evaluate the engagement and acceptability of OptimalMe, a digital preconception intervention. Participants, (*n* = 298) Australian women aged 18–44 with private health insurance planning to conceive within 12 months, received a standardised intervention, including access to a digital healthy lifestyle platform (educational materials, behaviour change activities, and self-monitoring resources), ongoing text messaging, and remotely delivered health coaching (two appointments) with randomised delivery methods (telephone/videoconference). Engagement and acceptability were assessed through mixed method analyses. The results show that 76.2% attended both coaching sessions, with similar participation rates for telehealth (75.2%) and videoconferencing (77.2%) (*p* = 0.469). All participants logged into the digital platform, with 90.6% accessing educational materials and 91.3% using behaviour change tools. Digital platform engagement declined over time, suggesting potential benefits from additional health coaching support for ongoing participation. The post-intervention evaluation (*n* = 217 participants) demonstrated that approximately 90% found the digital module engaging, meeting information needs, would recommend the program, and were satisfied with the support. OptimalMe demonstrated positive acceptability and engagement; however, further research is warranted to explore strategies for sustaining engagement with the digital interventions.

## 1. Introduction

Optimising health and lifestyle behaviours during the preconception period offers a pivotal window for enhancing maternal, foetal, and neonatal health outcomes [1], particularly in pervasive obesogenic environments, where adverse lifestyle behaviours such as excessive energy intake, poor diet quality, and suboptimal physical activity lead to accelerated weight gain [2,3]. In Australia and internationally, the rise in weight among reproductive-aged women has become a notable trend, raising concerns about its potential impact on women’s health and reproductive outcomes [4,5]. Nutrition and lifestyle behaviours including folate supplementation, smoking and alcohol cessation, and preventative medical screening and review are also key to preconception health [6]. These opportunities underscore the need to reach and engage women across the reproductive continuum from preconception, into pregnancy, and post birth [7]. Despite this, fewer than half of women planning a pregnancy consult a healthcare provider for preconception care (PCC) [8], and of those that do, less than 20% report receiving healthy lifestyle guidance [9]. The low uptake of PCC is influenced by factors across individual, social, and health system domains. Individual barriers encompass a lack of awareness and a perceived low need for PCC [10,11,12]. Socially, limited awareness among the population and peers, coupled with the stigma surrounding sexual and reproductive health, discourages PCC seeking [10,11,12]. Health system factors involve healthcare providers’ inadequate knowledge and confidence, along with resource constraints such as time and funding [12]. Additionally, the under-integration of PCC into healthcare systems leads to missed opportunities for early intervention and education. Addressing these diverse barriers is essential to improving PCC utilisation and promoting healthier pregnancies and offspring.

In recent years, digital health interventions have emerged as a promising avenue for enhancing women’s health, demonstrating an ability to more readily reach, engage, and provide support during preconception [13] and pregnancy [14,15]. Digital health technologies encompass a range of tools such as mobile applications (apps), wearable devices, and web-based platforms offering a convenient and accessible means of delivering personalised health information, lifestyle support (i.e., nutrition, physical activity, and weight), and self-management opportunities [14,16]. Moreover, the integration of remote coaching within digital interventions has further expanded the potential for personalised support. This approach involves the delivery of health and lifestyle coaching through virtual platforms, including telephone and videoconferencing, enhancing the accessibility and flexibility of intervention delivery. Mobile apps and web-based platforms offer additional benefit, with the ability to facilitate social networking or peer support and deploy interactive tools, thereby enhancing potential for engagement and behaviour change support [17,18,19]. Despite the opportunities afforded by digital health, there is little known about the associated uptake and engagement of digital healthy lifestyle interventions in preconception, particularly within otherwise healthy, general populations. 

To address this research gap, we developed the OptimalMe program [20], a digital program adaptation based on extensive evidence from face-to-face-delivered lifestyle interventions. OptimalMe is a healthy lifestyle program providing education, support, resources, and tools, supported by personalised health coaching and messaging, designed to improve lifestyle behaviours and optimise prevention. The current study outlines a secondary analysis of the OptimalMe program to understand the level of engagement with and acceptability of program components, as well as factors influencing the engagement and acceptability of digital health interventions in the preconception period.

## 2. Methodology 

### 2.1. Intervention Methodologies

#### 2.1.1. Intervention Overview

OptimalMe is a type 3 hybrid effectiveness–implementation study with its detailed methodology and recruitment processes comprehensively described elsewhere [20]. The trial design aimed to generate key implementation learnings to inform the feasibility of future scale up. The program was delivered and implemented in partnership with Medibank Private, Australia’s largest provider of private health insurance, as previously reported [20]. Medibank invited members who had joined with, or upgraded to, pregnancy and birth insurance cover within three months, as a proxy for future conception. A co-designed process was developed with the implementation partner to facilitate Australia-wide recruitment using an opt-in design. Medibank Private had no role in the intervention design, outcome measures, or data analysis and reporting. Eligibility criteria were minimal, in line with the implementation design, including women who were not pregnant, yet intended to conceive within 12 months at point of recruitment, aged 18 to 44 years, of any BMI, who read and spoke English, with access to an internet-capable device. The parallel, two-arm, randomised trial design included randomisation at the level of the individual by a study biostatistician using a computer-generated list. Here, we report on engagement and acceptability outcomes during preconception intervention delivery. 

#### 2.1.2. Delivery and Implementation 

All participants received the same evidence-based, individually delivered, lifestyle intervention comprising access to the OptimalMe digital platform, two preconception health coaching sessions, and monthly text messages. Participants were randomised to different health coaching delivery modes (telephone or videoconference), allowing for a comparison of the implemented delivery methods. The intervention aimed to improve participant’s self-management capacity and healthy lifestyle through skill development and support including improving knowledge, goal setting, problem solving, and relapse prevention, underpinned by the self-determination theory and motivational interviewing [21]. 

The intervention was designed to reach women outside of primary care, cater to different learning styles and preferences, and to maximise cost-effectiveness via remote and digital delivery, whilst retaining personal contact and individualised content. One-on-one coaching sessions provided an opportunity to evaluate personal health priorities and practice personalised self-management skills including goal setting, self-monitoring, nutrition, and physical activity advice and to enable an evaluation of health coaching engagement and acceptability. The first coaching session occurred 1–2 weeks after enrolment, and the second 6–8 weeks after the first session. 

The first session was used to discuss the participant’s personalised digital PCC checklist, to encourage pregnancy preparation and increase awareness of preconception health topics. The personalised checklist was generated using the participant’s baseline lifestyle behaviours and relevant preconception health information, captured in a digital platform via survey during the onboarding process, as described previously [22]. Information was provided on simple lifestyle changes and the building of their behavioural self-management capacity including action planning, identifying and addressing obstacles, and relapse prevention. At the end of the initial one-on-one session, participants had generated one or more healthy lifestyle goals and an action plan based on self-defined priorities. If the participant had not utilised the digital goal-setting tool before or during the coaching session, healthy lifestyle goals were verbally discussed, and an overview of the goal/s was emailed by the coach to the participant at the completion of the session. Participants were encouraged to review their goals every two weeks. 

The second coaching session was used as an opportunity to review PCC actions and goal progress and to assess engagement with the digital platform. If participants did not attend their session after two phone calls or after waiting 10 min in the videoconferencing platform, they were contacted via email to reschedule. Participants were rescheduled up to two times. Those who did not attend their one-on-one session were emailed a summary of the core messages provided. All participants received the same, generally healthy lifestyle text messages, which reinforced the program messages and accountability.

### 2.2. Intervention Components 

#### 2.2.1. OptimalMe Digital Platform

The intervention protocol provides extensive details about the digital platform and tools [20]. In brief, the digital interface of OptimalMe comprises three elements, each fulfilling unique yet interconnected purposes: a health and behaviour assessment for PCC, an educational resource, and a personalised goal setting and progress monitoring tool. Each of these elements was designed to adapt and cater to the specific requirements of individual participants. 

#### 2.2.2. Health and Behaviour Assessment for PCC 

Baseline quantitative questionnaires assessed health and lifestyle outcomes to compile a personalised checklist of PCC action items [22], and to present educational factsheets in order of relevance to each individual. The questions assessed reproductive history, genetic/family history, physical assessment (weight, height, chronic diseases, cervical screening history), immunisation status, supplementation (folate/folic acid and iodine), contraception use, self-weighing frequency, dietary habits, physical activity, sedentary behaviours, and substance use (tobacco, alcohol, recreational drugs).

#### 2.2.3. Educational Resources

The educational component includes informative factsheets covering a range of health and lifestyle topics related to PCC and offers guidance on maintaining a healthy lifestyle to prevent weight gain, aligning with national dietary and physical activity guidelines [23,24]. Each factsheet was designed with a concise description and accompanying visuals. Upon accessing a factsheet, participants were presented with an optional quiz question at the beginning, encouraging engagement and assessing their knowledge.

At the conclusion of each factsheet, a clickable button enabled participants to include or exclude the corresponding action item on their personalised PCC checklist. This checklist was generated based on participant’s initial self-reported health status and behaviours, and could be modified upon interaction with the factsheets, allowing participants the flexibility to generate a checklist that reflects their specific priorities and perceived needs after reviewing the information. The checklist was included on participant’s digital profile, allowing participants to mark tasks as completed. 

#### 2.2.4. Personalised Goal Setting and Progress Monitoring Tool

The behaviour change component included an interactive, digital goal-setting tool. The tool allowed participants to select from a list of pre-defined health or lifestyle goal areas, and then complete a step-by-step process to develop a SMART goal and set an action plan [25], which was stored in the participant’s digital profile and could be reviewed, refined, or marked as completed. Developed action plans included the identification of a goal, associated barriers and enablers, and strategies to overcome barriers, as well as individually identified timeframes to practice the developed goal. Pre-defined health and lifestyle goal areas included, but were not limited to, drinking soft drink or sugary drinks, drinking water, alcohol consumption, making health a priority, weight, eating regular meals exercise, time spent sitting, time spent relaxing, sleep, and work/life balance. A review function enabled participants to continually reassess their goals or objectives via a text-based summary or a scaled evaluation out of ten. 

### 2.3. Data Collection and Evaluation Methodologies

A summative evaluation investigated the overall program effectiveness, usefulness, acceptability, and individual self-reported outcomes. Mixed method data collection was utilised, recognising the synergistic benefits of both quantitative and qualitative research methods. The data collection included (1) quantitative participant questionnaires completed at baseline and evaluation, (2) qualitative semi-structured interviews, (3) OptimalMe digital platform data, and (4) program checklists and observations completed by the research team, with the associated methodologies detailed below.

**Figure 1:**
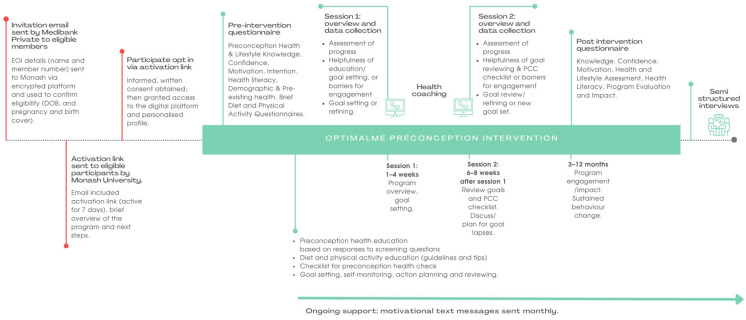
OptimalMe preconception intervention overview.

Figure 1 outlines the program overview, including the intervention components and data collection. 

### 2.4. Outcome Measures

#### 2.4.1. Baseline Questionnaire

Demographics, reproductive health, and lifestyle factors were captured at baseline, as previously reported [22]. Self-reported weight and height were used to calculate BMI (weight/height (m^2^)), which was classified according to the World Health Organization’s definitions [26]. Additionally, reasons for participating and program expectations were captured using a 5-point Likert scale to ascertain how strongly participants agreed with the following statements: ‘I want to manage my weight; I want credible information about how to prepare for pregnancy; I want expert health support prior to pregnancy; I want to be more informed about health prior to pregnancy; and, I want to increase my chances of becoming pregnant’. 

#### 2.4.2. Evaluation Questionnaires

A post-intervention evaluation and reminders were sent to all participants via an electronic link, sent by email and text message, commencing three months after enrolment, following preconception intervention dose completion (the completion of two coaching sessions, or receiving core messages via email for non-attendants). The questionnaire was developed by the project team, adapted from previous program evaluations [27]. Five-point Likert scales were used to measure engagement, helpfulness and likelihood of recommending the program to others. Participants were asked the following: ‘how engaging did you find the online module’; ‘how helpful were the following: overall digital platform; physical activity, diet and weight factsheets and resources; preconception health factsheets; health and lifestyle coaching; text messages, and self-weighing’ and ‘how likely are you to recommend OptimalMe to family and friends thinking about becoming pregnant?’. A higher score indicated greater satisfaction or acceptability. 

Multichoice and binary questions were used to further assess satisfaction and on-going intervention engagement. Participants were asked if the module met their information needs and if they were satisfied with the level of support provided by the research team (yes or no and a free-text option), and the frequency (daily, weekly, monthly, or occasionally or never), and duration of time (less than 10; 10–30; more than 30 min) that they typically logged on for if/when engaging with the OptimalMe platform. 

#### 2.4.3. Semi-Structured Interviews 

The acceptability of each program component was assessed through in-depth qualitative semi-structured interviews, conducted after the intervention, in a sub-group of participants. All women who had received the intended intervention dose at the time of the interviews (Q3 and Q4 2021) were invited to participate via email. After the initial email invitation, recruitment reminder emails were sent to all eligible participants alongside conducting interviews to recruit participants until saturation occurred. One trained researcher conducted all interviews (L.M.), guided by an interview schedule that was modified from previous program evaluations assessing acceptability [27] (Supplementary File S1). The interview guide focused on seven broad topics: (1) participation and expectations, (2) digital platform usability and appearance, (3) goal setting interactivity and engagement, (4) information/program delivery, (5) health and lifestyle coaching, (6) behaviour change and impact, and (7) satisfaction and improvements. 

Those who agreed to participate provided informed consent and were interviewed by phone for approximately 30 min. Interviews were conducted until data saturation was met, determined when no new ideas emerged from the interviews, as per standard methods [28]. All qualitative semi-structured interviews were audio-taped and transcribed verbatim by a professional service. 

#### 2.4.4. Digital Platform Data 

Participant interaction with and use of digital platform components such as the information factsheets, quiz questions, and the goal-setting tool were used to assess engagement with the OptimalMe digital platform. Timestamped digital platform generated user data were collected in Dynamo DB database tables saved on the Monash University Amazon Webservices (AWS) backend of the application. An interaction with a factsheet was generated if a participant opened an article. Quiz questions could be answered only once; the user had to select their response and then ‘submit’ their answer. Each stage of the goal setting was captured (selecting a goal area, manually transcribing and setting a goal, and reviewing progress with a goal) and retained on the backend if the goal was marked as complete. 

#### 2.4.5. Program Checklists and Observations

Devised program specific checklists and process data were developed to evaluate the program fidelity and researcher evaluation. Fidelity checklists documented the completion of intervention components by the participant at the time of the one-on-one coaching sessions (i.e., reading factsheets and using behaviour change tools), and participant engagement with session deliverables (i.e., discussing the PCC checklist, diet and physical activity guidelines, and goal setting), and participant perceptions of digital content and tools, such as ease of navigation, self-reported use, and relevant barriers and enablers for engagement. A verbal response was collected by the coach and documented in the checklist during the one-on-one session. 

## 3. Analyses

### 3.1. Quantitative

A data analysis was conducted using SPSS version 27.0 for Windows. The results were presented as mean (SD) for continuous and relative frequencies for categorical data. A *p* < 0.05 was considered statistically significant. Where a significant *p*-value was identified in a multiple comparison, Bonferroni correction was used to examine if the significance remained after adjusting for multiple groups [29]. All descriptive statistics were tested for normality using the Shapiro–Wilk test. 

### 3.2. Qualitative 

De-identified transcripts were coded (NVivo Software program, QSR International Pty Ltd., Version 11, Melbourne, Victoria, Australia) and thematically analysed independently by two investigators (B.R.B. and C.M.). In depth discussions of emerging themes took place before a final iteration of the results was agreed upon between investigators. Independent researchers with no involvement in the development or delivery were included to conduct interviews (L.M.) and assist with analysis (C.M.). Grounded theory principles guided the analysis, enabling the identification, coding, and categorisation of primary data patterns [30]. 

### 3.3. Triangulation

Quantitative and qualitative analyses were conducted separately, and then a triangulation of findings from different methods was conducted when interpreting the results. Qualitative and quantitative findings from various components of the study were compared to determine where findings from each method agreed (convergence), offered complementary information on the same issue (complementarity), or appeared to contradict each other (discrepancy or dissonance) [31]. 

## 4. Ethics

The Monash Health Human Research and Ethics Committee approved the study (date: 4 September 2019, reference: RES-19-0000291A), which has been registered on the Australian and New Zealand Clinical Trial Registry (ACTRN12620001053910). Participants provided written, informed consent to take part in the study. 

## 5. Results

Overall, *n* = 298 were enrolled in OptimalMe, with *n* = 153 randomised to telephone and *n* = 145 to videoconference coaching. Participants had a mean (SD) age of 31.8 (4.3) years and BMI of 25.7 (6.1) kg/m^2^. No significant baseline differences in demographic characteristics were found between the health coaching groups, as previously reported [22].

Of the enrolled participants, *n* = 217 completed the evaluation questionnaires, equivalent to 72.8% of the study population, an average of 4.5 months after commencing the intervention. Using demographic information, some evaluation data (27%) were found to be missing completely at random (*p* = 0.112); therefore, the imputation of missing data was negated. 

In total, 31 (*n* = 16 still in preconception, *n* = 15 pregnant at time of interview) participated in the semi-structured interviews. The mean (SD) age and BMI of the interview participants were 31.7 (4.0) and 25.5 (9.6); all completed the completed the evaluation questionnaires, and 93.4% (*n* = 29) attended both preconception coaching sessions.

**Figure 2:**
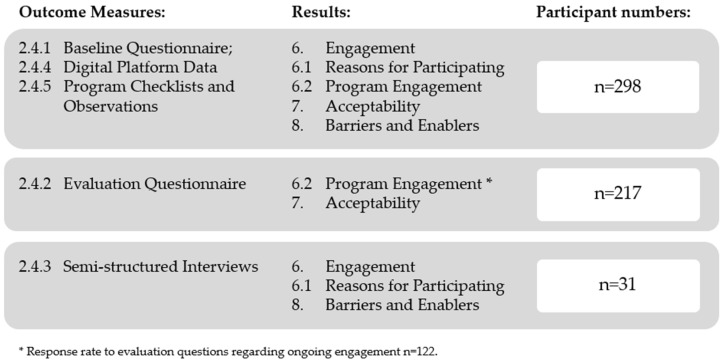
Outcome and evaluation of response rates.

Response rates for outcome measures and result sections are displayed in Figure 2.

## 6. Engagement

### 6.1. Reasons for Participating 

Most participants joined the program for reasons relating to pregnancy preparation and access to relevant and credible information. All women (100.0%) agreed or strongly agreed that their reason for joining OptimalMe was to obtain credible information about how to prepare for pregnancy (*n* = 58, agree, and *n* = 239 strongly agree), and that they wanted to be more informed about health prior to pregnancy (*n* = 80, agree, and *n* = 217 strongly agree). Almost all (99.7% and 99.3%, respectively) joined to access expert health support prior to pregnancy to increase their chances of becoming pregnant. Over 80% (*n* = 242/297) joined because they wanted to better manage their weight. 

Themes emerged from the semi-structured interviews that converged with baseline quantitative findings, such as pregnancy preparation, motivation to improve health prior to pregnancy, and a desire to improve preconception knowledge:

‘I was … just starting to explore the idea of trying to conceive and I was … thinking it was probably something that I didn’t have a lot of background knowledge in. I’ve had lots of friends who have had kids, so I’ve got their anecdotal information, but I was quite conscious of actually [personally] knowing very little about women’s health’.

(OM312, Videoconferencing)

‘I thought it would be a good opportunity to learn something that I had no knowledge on; an area that I didn’t know much about and was just really keen to get as much info as I could on this journey’.

(OM085, Telephone)

Additional complementarity qualitative themes around health improvement most often emerged in participants who noted health concerns or previous pregnancy experiences:

‘I just wanted to make sure I was the healthiest that I could be before I got pregnant [after the loss of our newborn]’.

(OM182, Telephone)

‘I thought it would be a good chance—cos we’re looking at getting pregnant again—to get some healthy habits in place before or while on the journey to having another baby […] I’ve got three other kids’.

(OM112, Telephone)

The quiz questions were an effective way of increasing user engagement with topics or content:

“‘The structure of [the factsheet] was good, because it had those true and false (quiz) questions at the start, where you can test your own knowledge. If I got one wrong, I was quite surprised myself, [and thought]’ okay maybe I can read into this a little bit more, why did I make that choice of that answer?”.

(OM013, Videoconferencing)

### 6.2. Program Engagement

All enrolled participants completed the first preconception coaching session. Engagement with both preconception coaching sessions was 76.2% (*n* = 227) overall (75.2%, *n* = 115 telehealth and 77.2%, *n* = 112 videoconferencing, *p* = 0.469). All women had access to the digital platform, logged in at least once, and were sent between 14–26 text messages, depending on their time of recruitment. 

At the point of the first session, approximately 70% of participants (*n* = 211/298) accessed the platform 1–2 times. Less than 10%, (7.0%, *n* = 21), had not accessed the platform before attending their first coaching session, and 22.1% had logged in three or more times. 

During the intervention, digital factsheets relating to preconception health and lifestyle were accessed by 90.6% (*n* = 270), with most (82.6%, *n* = 246) accessing multiple factsheets. Fertility (74.5%, *n* = 222), vaccinations (65.8%, *n* = 196), and genetics (63.4%, *n* = 189) were the most highly accessed topics. Overall, *n* = 2634 factsheet interactions were recorded, across a total of 17 preconception health and healthy lifestyles topics, equating to each factsheet being accessed 154 times across the 298 participants. Most participants (82.9%, *n* = 247) engaged with factsheet quiz questions, generating a total of *n* = 2163 quiz responses. 

The digital platform backend data demonstrated that the goal-setting tool was utilised by 91.3% of participants, with *n* = 272 selecting one health or lifestyle goal area to address, and 76.5% (*n* = 228) completing one or more goals and identifying their personal obstacles and strategies. The most commonly selected goal areas were exercise (66.8%, *n* = 199), weight (51.7%, *n* = 154), and time spent sitting down (43.3%, *n* = 129). 

The platform backend data demonstrated that self-monitoring using the digital goal review tool was utilised by 21.1% (*n* = 63/298), who reviewed their progress with at least one goal. During the second coaching session, most participants self-reported to the coach that they had reviewed action items on their PCC checklist (72.2%, *n* = 164/227). Approximately 80% (*n* = 178/227) reported reviewing their goal and progress independent of the digital platform, while 35.7% (*n* = 81/227) had started working on another goal in addition to their original behaviour change domain. 

Of those who attended the second session (*n* = 227), most had logged on 1–2 times (60.8%, *n* = 138/227) between the first and second session. Approximately a quarter of those who attended the second session (22.2%, *n* = 50/227) had not logged in at all between sessions. However, 58.0% (*n* = 29/50) of those who had not logged into the digital platform had still considered or actioned elements of their PCC checklist, and 78.0% (*n* = 39/50) had passively thought about their goals. 

At evaluation, 77.0% (*n* = 94/122) reported that they were still logging into the digital platform; however, most were doing so occasionally (50.8%, *n* = 62/122) or monthly (22.1%, *n* = 27/122). Approximately one quarter (23.0%, *n* = 28/122) were not logging in at all, and responses were missing for 18.9% of evaluation respondents (*n* = 41). When logging in, the majority (54.1%) were logging in for 10 min or less, or 10–30 min (42.9%). No significant differences were observed between delivery groups (*p* = 0.253 for frequency, *p* = 0.712 for time).

## 7. Acceptability

At the time of the first session, participants reported to the coach that the digital platform was easy to navigate (mean score of 8.6/10), which was verbally ascertained and recorded via the session checklist. Evaluation demonstrated high levels of overall program acceptability (considered to be a response of 3–5) with 90.3% (*n* = 196/217) responding, upon evaluation, that the digital module was engaging, and 89.9% (*n* = 195/217) felt the module met their information needs. Overall, 90.8% (*n* = 197/217) reported recommending the program if it were available to friends or family planning a pregnancy, and 90.7% (*n* = 195/215) were satisfied with the level of support provided by the research team. Those in the videoconference coaching group were significantly more likely to recommend (*p* = 0.042) the program to others planning a pregnancy, with no other significant differences found for specific acceptability factors between groups. The majority of participants found the information or program components to be helpful. The most helpful component was health and lifestyle coaching (91.7%, *n* = 198/216), followed by the preconception-specific health factsheets (90.7%, *n* = 195/215), physical activity, diet and weight factsheets and resources (90.3%, *n* = 195/216), information about weight gain prevention and regular self-monitoring, including an embedded BMI calculator (74% *n* = 159/215), and regular text messages (72% *n* = 153/213). Suggestions for program improvements included the addition of meal plans (46.1%, *n* = 100/217) and exercise plans (40.6%, *n* = 88/217) as well as more health coaching sessions (35.0%, *n* = 76/217), social media groups for program participants to interact with and provide encouragement to each other, and more text messages (20.7%, *n* = 45/217).

## 8. Barriers and Enablers 

Factors influencing engagement and acceptability were documented via checklists completed during coaching sessions. The main barrier for engaging with the education resources (factsheets) was ‘lack of time’. The main barriers for engaging with the goal setting function were ‘lack of time’, and ‘not knowing how to complete the goal setting task’. Other reasons noted were, ‘waiting for the one-on-one session’, ‘wanting support’, and finding the task ‘overwhelming’ or ‘hard to complete’. 

Within semi-structured interviews, themes emerged that complement the findings for acceptability and engagement:

Theme: desire for support from the health coach to facilitate goal setting for enhanced confidence, motivation, and accountability regarding their goals:

‘you’ve got to make sure the goals are achievable and reasonable […] you can so quickly be demotivated if you just don’t feel like you’re getting anywhere with your goals setting. So, I think going through it with someone else and talking strategies and sort of getting a check that each goal is sort of reasonable and achievable is really important’.

(OM112, Telephone)

‘on the website, [it wasn’t] the biggest motivator for me; it was more talking to [the coach] and going through those goals, which is basically just a verbal version of that. But to have feedback from another person about what is a realistic goal and what’s not a realistic goal was really useful’.

(OM331, Videoconferencing)

‘The first ones that I did were about increasing exercise and water intake. For those, it felt really easy to work out what would my goal be and realistic steps to achieve that goal. But if it was more specific, or a bit more niche, then it would probably be a bit more difficult, so I guess the broad stroke goals that all women would have are pretty easy to do, but some of them are more specific to each person and might be a bit more difficult’.

(OM331, Videoconferencing)

Theme: desire for increased functionality of the digital platform that would enhance user experience and increase program interactions. 

Numerous women suggested a mobile app and that the associated features, such as push notifications, would have increased engagement:

‘some way of it popping up [goal reminders]. [Such as] it’s been, however long since you’ve started this goal, how are you finding you’re going, or […] something like that’.

(OM227, Telephone)

‘if it was an app, you could have notification or things to say ‘have you checked your goal this week or something?’ Yeah, I don’t know little things like that [would encourage me to keep] logging on and checking in’.

(OM182, Telephone)

‘there was probably [room for] improvement, in terms of it prompting you to go to the next section, […] it would be good if there was a bit of a like a workflow that would prompt you to say … instead of asking you to click it, it would almost just appear, so that you keep going [through the education content or tools]’.

(OM202, Videoconferencing)

‘[I’d prefer a mobile app] because I don’t have to go on to a website [and] sign in; I just have to click on to an app, probably touch my finger for password protection and then I can just click, rather than get to a computer, go online and do it. Rather than just have something that’s in my pocket that I could be doing while I’m travelling to work’.

(OM190, Telephone)

## 9. Discussion

This study evaluated the engagement and acceptability of OptimalMe, a digital preconception health lifestyle program featuring individual health coaching and ongoing behaviour change support [20]. Low engagement with PCC and suboptimal lifestyle behaviours before pregnancy [1,8,9,22] underscore the need for innovative solutions. This research addresses this gap by exploring the potential of digital healthy lifestyle programs, offering promise in reaching and engaging women intending to conceive. These programs aim to improve knowledge, encourage behavioural changes, and enhance health outcomes before pregnancy. Understanding factors that drive intervention acceptability and engagement can optimise intervention design and, in turn, enhance their effectiveness. Here, we report that pregnancy preparation, access to credible information, and the desire to improve health prior to conception were primary motivating reasons to join OptimalMe. The most valued intervention component was personalised coaching, and encouragingly, delivery via telephone or videoconference did not alter engagement or acceptability. Intervention components supporting the individualised health coaching and the digital platform were highly accessed, but with declining engagement over time. Overall, these results emphasise the importance of personalised, individual coaching as a cornerstone of healthy lifestyle intervention designs with flexibility in delivery methods to maximise program acceptability. These results demonstrate that future research is required to further develop, enhance, and evaluate digital technologies as supporting intervention resources. 

Program engagement refers to the level of involvement, participation, and interaction of individuals with a specific program or intervention. Detailed insights into engagement are important as it directly influences program retention, satisfaction, behaviour change, and overall effectiveness and impact, whilst providing insight for future improvements. Yet the term engagement is broad and measured variably, yielding inconsistent findings and creating difficulty in synthesising reliable measures [32]. For health interventions, this may be due to the complexity of designs and the inclusion of numerous components. This study considers engagement in line with behavioural sciences, which interprets engagement objectively, focusing on the frequency, duration, and depth of intervention component usage [33]. Engagement, in turn, is influenced by intervention acceptability, which also predicts key outcomes such as intervention effectiveness and adoption, as participants are more likely to adhere to, and benefit from, an intervention if it is acceptable [34,35]. In the human–computer interaction literature, acceptability considers the cognitive and affective state of a participant’s flow in a program, encompassing absorption, immersion, and enjoyment [36]. While this can be measured using sensor data and psychophysiological measures, here, we drew on self-reported acceptability to derive insights into absorption and enjoyment. The majority of participants in the OptimalMe program had positive experiences with the digital platform. They found it to be informative, engaging, and user friendly. Additionally, they indicated a willingness to recommend the program to their family or friends. These findings suggest that the program was highly acceptable to the participants and appeared to meet the needs of the target population through the provision of reliable resources. This alignment between the intervention’s goals and the participants’ needs is crucial for the success and impact of any healthcare or educational program.

As an objective measure, the engagement levels within various components of the OptimalMe program were noteworthy. Both the education-based component and behaviour change component of the digital platform had high levels of participant engagement, alongside health coaching. Participants actively utilised platform components, with almost all participants accessing information factsheets, interacting with quiz questions and the PCC checklist, and completing behaviour change activities, including goal setting. The most accessed topics were fertility, vaccinations, and genetics in line with the primary reasons for program participation, including the seeking of knowledge and support in preparation for pregnancy. These findings align with those of previous studies demonstrating that intention to conceive improves uptake of preparatory behaviours [1]. However, interaction with the digital platform and tools declined over time, with approximately a quarter not logging in between coaching sessions, and the majority reporting that they had logged in once or twice. Upon evaluation, most participants reported logging in occasionally or monthly, while one quarter were not logging in at all. Given that the individual health coaching was the most valued intervention component and was shown to enhance engagement with the digital platform, it is likely that the completion of the health coaching impacted ongoing engagement with supporting intervention components. Alternatively, it is plausible that at the completion of health coaching and at the point of evaluation, participants had already accessed or engaged with the content they perceived as most valuable, as well as actioned their PCC checklist, thereby negating the need for frequent re-engagement. Furthermore, parts of the behaviour changing, goal setting, and action planning require time for adoption, reinforcement, habit formation, addressing contextual factors, and overcoming resistance [37]. This was a primary component of health coaching, and therefore, at completion, participants may have felt more able to practice and review goal setting without the assistance of the digital platform, as similarly demonstrated in our previous HeLP-her studies utilising program manuals and paper-based activities to support health coaching [27].

The desire for personalised support and guidance in behaviour changing emerged as a significant factor influencing both engagement and acceptability. Some participants reported challenges in completing goal-setting activities autonomously and valued the role of the health coach in setting achievable and realistic goals. Participants sought feedback and validation from the coach, emphasising the importance of human interaction in goal setting, motivation, and ultimately behaviour changes toward healthy lifestyles. This highlights the need for digital interventions to incorporate tailored support and personalised coaching to enhance participant engagement. In line with our findings, personalised support has previously been shown to be of higher value over other program components such as educational resources [27]. Personalised interventions offer greater ability to address enablers and barriers to behaviour change [38], and health coaching has been shown to lead to significantly better outcomes for weight management [39]. The personalisation of tailored guidance, motivation, and feedback can cater to individual’s unique needs, preferences, and circumstances, which appears to be important for addressing participant’s lifestyle-related barriers. This individualised approach enhances participants’ sense of autonomy, accountability, and self-efficacy, leading to better adherence to behaviour change goals [38]. In OptimalMe, the health coaching appeared to enhance the acceptability of the goal setting, compared with the presentation of the behaviour change tool on the digital platform. Participants reported not easily understanding the digital goal setting activity, and many did not review their goals digitally, but did so in the coaching session. This indicates the digital platform was less acceptable in facilitating ongoing behaviour changes or maintenance activities. Further research is needed to determine methods and tools that retain engagement with digital health interventions and encourage self-monitoring. Integrating personalised support and individual coaching in digital and lifestyle interventions appears to be crucial for optimising participant engagement, motivation, and ultimately improving intervention effectiveness.

While insights pertaining to both engagement and acceptability were consistent between coaching delivered by telephone and videoconference, there was a significant difference for program recommendation. Individuals who received video coaching were more inclined to express their intention to recommend OptimalMe to their family and friends, potentially indicating that the visual and interactive nature of videoconferencing may have a greater impact on participants, thereby influencing their likelihood to endorse the OptimalMe program. This suggests that the mode of coaching delivery can play a crucial role in the perceived value and endorsement of the intervention among reproductive-aged women. This preference may also signify that video-based interactions cultivate a stronger sense of trust in the overall program’s reliability and effectiveness, reinforcing confidence in its capacity to deliver positive results. Further exploration of these nuances can contribute to refining digital interventions with embedded coaching to better align with the preferences and communication styles of the target population. 

Effective health coaching is invaluable for improving accountability and engagement among participants. The personalised guidance, support, and accountability provided by human coaches play crucial roles in helping individuals make meaningful progress toward their health goals, and therefore, human-centred coaching remains a cornerstone of successful health intervention [27,38,39]. However, coaching is not a standalone solution, and if complemented by a well-designed digital platform, the overall experience and impact can be enhanced. The digital platform can reduce the burden on coaches and also strengthen their efforts by making the coaching experience more dynamic and comprehensive. To improve the digital platform, participants have suggested several key enhancements, such as more personalised features, including meal plans and exercise plans. Tailoring content to individual needs could enhance user engagement and make the platform more effective in supporting coaching efforts to improve lifestyle behaviours. The effectiveness of quiz questions in increasing engagement with the platform’s content suggests that incorporating more interactive components may make learning more engaging. Finally, the desire for a digital platform that delivers push notifications underscores the importance of platform accessibility and timely reminders to sustain user engagement and involvement in behaviour change objectives. The suggested improvements align with features and tools commonly found in popular commercially developed mobile apps for the perinatal period [40]. High quality commercially developed apps appear to be highly engaging and acceptable to women during their reproductive journey; however, health and lifestyle information or behaviour change tools within them are often of poor quality [40]. Therefore, partnerships among consumers, researchers, designers, developers, policy makers, and the health care sector may present an opportunity to provide women with co-designed intervention resources that balance the valued consumer attributes of apps, alongside evidence-based information and effective behaviour change techniques. Our findings highlight the importance of a user-centric digital platform that offers accessible, interactive, and engaging resources, maximising the program’s impact and reach. Combining a well-designed digital platform with effective health coaching in future interventions can enhance user engagement and offer opportunities for scalability and cost-effectiveness, expanding the reach of health promotion and preparation programs to a broader population.

## 10. Strengths and Limitations

Our rigorously developed questionnaires and numerous data collection methods have enabled deep insights into the autonomous, self-direct use of the OptimalMe digital tools and program, as well as research-directed insights such as through the evaluation and semi-structured interviews. The triangulation of these data provides novel insights in an under-researched preconception population. Utilising different data collection methods and timepoints has also developed a strong understanding about engagement over time, with acceptability concepts enabling insights into potential reasons for changes in engagement. The randomisation to health coaching delivery methods strengthens the understanding of the impact of remotely delivered health and lifestyle interventions on engagement and acceptability, and the methods and findings from this research can be applied and tested under other health or lifestyle topics, and in other populations. 

The results of this study need to be considered in light of its limitations. First, although women were recruited across Australia, participants were well-educated and from high-income groups, as reported previously [22]. This may limit the generalisability of the results and precludes essential insights regarding digital preconception health engagement and acceptability from women from diverse groups. Health coaching and evaluation attrition were similar in intervention groups; however, the post-intervention data collection did not include all participants, and the opt-in recruitment for semi-structured interviews may have led to the data collection preferencing participants who were more engaged with the intervention. Furthermore, the data collection took place shortly after the intervention, so long-term effects might be lower; however, further intervention insights from the ongoing intervention during pregnancy will address this limitation. The program was designed to be socially and culturally inclusive and to suit different levels of health literacy. However, the recruitment methods from this intervention did not enable us to test its impact in different groups. Further work is required to evaluate how the OptimalMe program meets the needs of LGBTQIA+, low-literacy, CaLD, and Indigenous people.

## 11. Conclusions

Our study highlights the importance of personalised health coaching in engaging women in digital health interventions when preparing for pregnancy. It also emphasises the acceptability and engagement with user-centric digital platforms to complement coaching efforts. While the study reveals positive engagement with the program, sustaining it over time remains a challenge. Participants valued human interaction for behaviour change objectives such as goal setting and motivation, underscoring the role of health coaching. The decrease in engagement with the digital platform following coaching could be attributed to factors such as the absences of ongoing coaching sessions, a lack of new personalised content, the perceived completion of essential tasks related to pregnancy preparation, or the ability to independently practice and review goal settings without relying on the digital platform. To enhance engagement with the digital platform, participants suggested additional personalised features, interactive components, and reminders. Combining effective coaching with an improved digital platform offers scalability and cost-efficiency for preconception health programs. Despite the study’s strengths, limitations include a lack of diversity among participants and potential data bias. Future research should address these gaps to better understand and enhance preconception health engagement and acceptability to improve preconception health.

## Data Availability

Data are available upon request to the corresponding author (C.L.H.). The data are not publicly available due to privacy.

## Supplementary Materials

The following supporting information can be downloaded at: https://www.mdpi.com/article/10.3390/nu16050572/s1. File S1: Semi-structured Interview Guide.
