# Supplementary material for: OptimalMe Program: A Mixed Method Investigation into the Engagement and Acceptability of a Preconception Digital Health Lifestyle Intervention with Individual Coaching for Women’s Health and Behaviour Change
Source: Nutrients. 2024 Feb 20;16(5):572. doi: 10.3390/nu16050572 (PMC10933940; doi:10.3390/nu16050572)
Supplement: Supplementary file 1 [file nutrients-16-00572-s001.zip › nutrients-2848956-supplementary.pdf]

Version 2, April 21st 2021, Semi-structured participant interview questions (pre-pregnancy, 3 months post enrolment - interview guide)

Semi-structured interview questions:

PARTICIPATION/EXPECTATIONS
1. To start off with, why did you decide to participate in this program?<br>*Probe: Preparing for pregnancy, well-being, lifestyle*
2. In your opinion what was the program about?<br>*Probe: what was your perception about the program purpose*
LOOK/FEEL/USEABILITY
3. Thinking about the Optimal website can you comment on the website's appearance and layout?
INTERACTIVITY/ENGAGEMENT WITH GOAL SETTING
4. As part of the program you completed goal setting. Did you find goal setting useful?<br>5. Did you review or revisit the Optimal Me website more than once?<br>*Probe:* If yes, what encouraged you to come back to the Optimal Me website? (reviewing and resetting goals/easy to understand/relevant to me)<br>6. Is there anything that is missing from the program?
7. Were you aware there was a pre-pregnancy health checklist available? Did you use this? Was it helpful?
INFORMATION DELIVERY
8. Having completed the pre-pregnancy program has your knowledge about pre-pregnancy planning and lifestyle choices improved? If so, can you describe the information that you have learned?
9. Do you feel that the amount of information provided was sufficient(right) to meet your needs?<br>- If not, what are you looking for? Can identify your needs? More information?<br>- Did the program answer all your pre-pregnancy and healthy lifestyle questions?<br>- Was the information too simple?<br>- Did you want something less self-directed and more prescriptive?<br>- Do you feel the program provided you with adequate support?<br>-
10. Of the delivery modes that were available (online information/text messages/health coaching), what did you enjoy the most? Why?
HEALTH COACHING (2 sessions) (80% women complete first session and 50% second?)
11. Were the one-on-one phone coaching sessions of value to you?<br>*If attended both phone coaching sessions*<br>Probe: If so why (support/rapport, knowledge/professional, accountability)?

If did not attend the second session<br>- Why not?<br>- Would a different format have been preferred? (face-to-face, Zoom or email)
12. Compared to completing the goal setting online on your own, did you find goal setting more valuable when completing it with the coach? Why?
13. Would you have preferred group or one on one sessions, or pre-recorded videos?
REACH/IMPACT/RECOMMENDATIONS TO OTHERS
14. made any changes to your food choices of physical activity since commencing this program? If so, what has changed?<br>- Have you been able to maintain this change?<br>15. Did you share with anyone your learnings from the program?<br>*Probe: Does anyone close to you know that you have completed the Optimal Me program? (Partners or friends)*<br>- Have you discussed lifestyle or pre-pregnancy health information from this program with anyone?
16. If a partner or support-person version of OptimalMe was available would this be something you think would add value?
SATISFACTION AND IMPROVEMENTS
17. Do you feel more informed after completing this program?<br>Probe: In your opinion do you believe this is a valuable and worthwhile program?<br>18. Is there anything that could have been done to make your experience better?<br>*Probe: would an online chat-forum with other women planning pregnancies or trying to conceive assist you?*
19. Are there any others comments you would like to make?
